# The association of the *Clock 3111 T/C *SNP with lipids and lipoproteins including small dense low-density lipoprotein: results from the Mima study

**DOI:** 10.1186/1471-2350-11-150

**Published:** 2010-10-21

**Authors:** Kokoro Tsuzaki, Kazuhiko Kotani, Yoshiko Sano, Shinji Fujiwara, Kaoru Takahashi, Naoki Sakane

**Affiliations:** 1 Division of Preventive Medicine and Diabetes Education, Clinical Research Institute for Endocrine and Metabolic Disease, National Hospital Organization Kyoto Medical Center, Kyoto, Japan; 2 Mima City National Health Insurance Koyadaira Clinic, Tokushima, Japan

## Abstract

**Background:**

The clock molecule plays major roles in circadian rhythmicity and regulating lipid and glucose metabolism in peripheral organs. Disruption of the circadian rhythm can lead to cardiometabolic disorders. The existence of small dense low-density lipoprotein (sdLDL) in the circulation, an abnormality of lipid metabolism, in part associated with lifestyle, is also one of risk parameters for cardiometabolic disorders. The *3111 T/C *single nucleotide polymorphism (SNP) of the *Clock *gene has been reported to be associated with lifestyle including morning/evening preference. We investigated whether the *Clock 3111 T/C *SNP may affect lipids and lipoproteins including sdLDL.

**Methods:**

In 365 community-dwelling subjects (170 men and 195 women, mean age 63 ± 14 years), the *3111 T/C *SNP was genotyped using a fluorescent allele-specific DNA primer assay system. The levels of sdLDL were measured with the electrophoretic separation of lipoproteins employing the Lipoprint system.

**Results:**

The frequency of the *Clock 3111 C *allele was 0.14. The area of sdLDL did not differ between the subjects with obesity and those without. In carriers of *T/T *homozygotes, the area of sdLDL was significantly higher compared with carriers of the *C *allele (*T/C *or *C/C*) (1.7 ± 3.4 vs. 0.8 ± 1.9%; p < 0.05). A multiple regression analysis showed that the area of sdLDL was significantly and negatively correlated with the *Clock 3111 T/C *SNP (β = -0.114, p < 0.05), independently of age, sex, body mass index, and exercise habits.

**Conclusion:**

Our findings indicated that the *Clock 3111 T/C *SNP might be associated with the existence of sdLDL.

## Background

Sleep can be interrelated with various lifestyle and genetic factors. The sleep-wake cycle is generated through circadian rhythmicity and homeostasis [1,2]. In mammals, physiological processes show approximate 24-hour rhythms [3] derived by the clock molecules controlled not only by the master circadian clock in the suprachiasmatic nucleus (SCN) [4-6] but also by peripheral clocks in the liver, muscle, and adipose tissue [7-9]. The clock molecule groups, such as brain and muscle Arnt-like protein-1 (BMAL1) and circadian locomotor output cycles protein kaput (CLOCK), also play major roles in circadian rhythmicity and regulating lipid and glucose metabolism in peripheral organs [10]. The human *Clock *gene located on chromosome 4q12 has a basic helix-loop-helix domains (for binding DNA). Mutant mice homozygous for *Clock *exibit an altered diurnal feeding rhythm, developing metabolic syndrome with hyperlipidemia [11], and a reduced amount of time spent asleep both in entrained and free-running conditions [12]. So, disruption of the circadian rhythm leads to metabolic and sleep disorders. A single nucleotide polymorphism (SNP), *3111 T/C*, located in the 3'-flanking region of the *Clock *gene was reported to be a predictor of diurnal preference in humans [13], although another study reported that the *Clock 3111 C *allele is not associated with eveningness [14]. The *3111 T/C *SNP influenced sleep and the activity patterns in people affected by bipolar depression, through a possible effect on the stability of mRNA of the *Clock *gene and the level of Clock protein [15].

Small dense low-density lipoprotein (sdLDL) is produced by abnormal lipid metabolism which leads to increased triglycerides (TG) levels and a difference in clearance between normal and abnormal TG-rich lipoproteins [16]. The existence of sdLDL in the circulation is associated with diabetes mellitus [17,18], diabetic nephropathy [19-21], metabolic syndrome [22], and coronary artery disease (CAD) [23,24]. SdLDL emerges, in part, through lifestyle and genetic factors [25-28]. In this study, we investigate whether the *3111 T/C *SNP of the *Clock *gene may affect lipids and lipoproteins including sdLDL.

## Methods

### Study subjects

All participants were recruited through an annual health check up in the Mima city, Tokushima prefecture in Japan. A total of 365 Japanese community-dwelling subjects, 170 men and 195 women; aged 24 to 88 years, were enrolled in this study. We included subjects who were asymptomatic without any known medical history of coronary heart disease and psychic disease. The study protocol was approved by the Ethics committee of National Hospital Organization Kyoto Medical Center. All the subjects signed an informed consent form after being fully informed about all aspects of the study before enrolling. After an overnight fast, body weight and height were measured using a body fat analyzer (OMRON. Co. Ltd., Osaka, Japan). The body mass index (BMI) was calculated as weight divided by squared height (kg/m^2^). The blood pressure was measured three-times at 10-minute intervals using a mercury sphygmomanometer. Venous blood samples were then drawn for blood tests. Blood glucose was measured by the hexokinase method (SHINO-TEST Corporation, Tokyo, Japan), and serum insulin was assayed by chemiluminescent immunoassay (Bayermedical.Co., Ltd., Tokyo, Japan). Serum total cholesterol (Wako Pure Chemical Industries, Ltd., Tokyo, Japan), high density lipoprotein (HDL)-cholesterol, and triglyceride (DAIICHI PURE CHEMICALS Co., Ltd., Tokyo, Japan) were determined by the enzymatic methods. Homeostasis model assessment of insulin resistance (HOMA-IR) was calculated according to a previous study [29]. Medical histories and lifestyle factors (exercise habits [none or done], sleeping time, skipping breakfast and smoking habits) were confirmed by a self-reported questionnaire and medical professionals' interview.

### Measurement of the area of sdLDL

The area of sdLDL was measured with the Lipoprint™ system (Quantimetrix Inc., Redondo Beach, CA) [30]. Briefly, 25 μL of serum sample and 200 μL of loading gel were applied to a 3.3% polyacrylamide gel tube and mixed well several times. Next, these samples were photopolymerized at room temperature for 30 min and then electrophoresed for 65 min (3 mA/gel tube). After the electrophoresis, a densitometric scanning was done with a ScanMaker i900 (MICROTEK Co., Carson, CA) and the lipoprotein subfractions were calculated with an iMac personal computer (Apple Computer Inc., Cupertino, CA). All the LDL subfractions were calculated based on a relative flotation rate (Rf) between the very LDL fraction as Rf = 0.0 and the HDL fraction as Rf = 1.0. LDL-1 and LDL-2 are defined as large LDL, and LDL-3 to LDL-7 are defined as sdLDL. The area of sdLDL is expressed as a percentage relative to total lipoproteins.

### Genetic analysis

A noninvasive method was implemented for collecting buccal mucosa cells using cytobrushes. After the phenol-extraction procedure, 0.2 to 2 μg of DNA was obtained. Genotypes were determined with an intercalater-mediated fluorescent allele-specific DNA primary assay (TOYOBO Co. Ltd., Tsuruga, Japan). The *3111C/T *polymorphic region of *Clock *(rs1801260) was amplified using the polymerase chain reaction with sense (5'-AAT ACC AGC CAG CAG GAG GTG ATC-3') and anti-sense (5'-CAA AAA ATA TCC AGG CAC CTA AAA CAC TG-3') primers, and labeled at the 5' end with Texas red (5'-ATA GGG GCA CAG CCA GTT C-3'). PCR amplifications were performed under the conditions recommended by the enzyme supplier. In brief, a 25-μL aliquot containing 20 ng of genomic DNA, the reaction buffer supplied, 3.0 mM MgCl2, 0.2 mM dNTP, and 1.25 U of rTaq containing anti-Taq high (TOYOBO Co. Ltd., Tsuruga, Japan). Cycling parameters were an initial denaturation at 95°C for 5 minutes, then denaturation at 95°C for 30 secconds, annealing at 65°C for 30 seconds, and primer extension at 72°C for 30 seconds for 40 cycles, and post extension at 72°C for 2 minutes in a thermal cycler (ABI9700). Two microliters of reaction mixture containing 10 pmoL of probe and SYBR Green (final concentration × 10000) was added to the PCR products. The mixture was placed in the ABI PRIZM 7700 and the melting temperature was measured. The program for analytical melting was 95°C for 30 seconds, then 40°C for 1 minute, increasing to 80°C by 10 minutes. The fluorescence signals were detected at excitation and emission wavelengths of 485 nm and 612 nm. In this study, all samples have checked by two independent investigators. No samples are observed different results.

### Statistical analysis

All statistical analyses were performed with the Statistical Package of Social Science (SPSS for Windows, version 11.0; SPSS Inc., Chicago, IL, USA). Data were expressed as means ± SD. The present study was designed to detect a difference in means equivalent to 1% of sdLDL with a SD of 3%. Thus, the sample size required was 286 with an error of 5%, with 80% power (beta = 20%) at the two-tailed 5% significance level. The differences in values between groups were estimated by using the Student's unpaired *t*-test and χ2-test. A stepwise multiple regression analysis controlled for measured parameters including the *Clock 3111 T/C *SNP was performed to explore the correlated factors with the area of sdLDL. P < 0.05 was accepted as statistically significant.

## Results

Table 1 shows the distribution of the *3111 T/C *SNP. The sex difference in the frequency was not observed (data not shown). The frequency of the *C *allele was 0.14, with 277 (75.9%) of subjects *T/T *homozygotes, 77 (21.1%) *T/C *heterozygotes, and 11 (3.0%) C/C homozygotes. No significant deviation from Hardy-Weinberg equilibrium was observed.

**Table 1:** Clinical and biological characteristics of subjects according to SNP of the *Clock *gene

Parameters	T/T	T/C	C/C	T/T + T/C	T/C + C/C	P value
Number	277	77	11	354	88	
Age (years)	64 ± 14	65 ± 13	61 ± 17	64 ± 14	65 ± 14	0.559
Sex (men/women)	132/145	31/46	7/4	163/191	38/50	0.464
Skipping breakfast (%)	5	9	0	6	8	0.390
Smoking (current, %)	14	25	27	26	16	0.249
Exercise habit (none/done, %)	72/18	70/30	54/46	71/29	68/32	0.061
Sleeping time (%)^a^						
< 6 hours	21.2	19.5	27.3	20.9	20.4	
6 hours	20.9	10.4	0	18.6	9.1	
7 hours	25.3	31.1	27.3	26.6	30.7	0.090
8 hours	24.2	33.8	27.3	26.3	33.0	
≥ 9 hours	8.4	5.2	18.1	7.7	6.8	
Body mass index (kg/m^2^)	24.0 ± 3.0	23.6 ± 3.3	25.8 ± 4.4	23.9 ± 3.1	23.9 ± 3.5	0.061
Systolic blood pressure (mmHg)	137 ± 19	134 ± 20	134 ± 21	136 ± 19	134 ± 20	0.228
Diastolic blood pressure (mmHg)	76 ± 12	76 ± 11	73 ± 15	76 ± 12	76 ± 11	0.735
Fasting plasma glucose (mmol/L)	5.7 ± 1.7	5.5 ± 1.9	5.9 ± 1.7	5.7 ± 1.7	5.6 ± 1.9	0.578
Fasting serum insulin (pmol/L)	47 ± 51	40 ± 29	35 ± 16	46 ± 47	40 ± 27	0.193
HOMA-IR	2.1 ± 2.9	1.8 ± 2.6	1.5 ± 0.7	2.1 ± 2.9	1.8 ± 2.5	0.331
Triglycerides (mmol/L)	1.13 ± 0.58	1.10 ± 0.51	1.00 ± 0.25	1.12 ± 0.57	1.09 ± 0.49	0.580
Total cholesterol (mmol/L)	4.84 ± 0.93	4.78 ± 0.88	4.29 ± 0.83	4.84 ± 0.91	4.73 ± 0.88	0.335
HDL-cholesterol (mmol/L)	1.42 ± 0.41	1.40 ± 0.36	1.40 ± 0.39	1.42 ± 0.39	1.42 ± 0.36	0.875
Small dense LDL (%)	1.7 ± 3.4	0.9 ± 2.0	0.3 ± 0.7	1.6 ± 3.2	0.8 ± 1.9	0.020

Data are means ± SD.

HOMA-IR, homeostasis model assessment of insulin resistance; HDL, high-density lipoprotein; LDL,

low-density lipoprotein

P values in a comparison between subjects with *T/T *genotype and those with *T/C *or *C/C *(by using Student's unpaired *t*-test and χ2-test).

In another model (*T/T *+ *T/C *vs. *C/C*), there were no significant differences in various traits (by using Student's unpaired *t*-test and χ2-test).

^a ^Four subjects with *T/T *genotype had no answer.

The physical characteristics and blood test results of the subjects are listed in Table 2. There were no significant differences between carriers of the *C *allele (*T/C *or *C/C*) and *T/T *homozygotes in gender distribution, age, BMI, systolic blood pressure, diastolic blood pressure, fasting plasma glucose, fasting serum insulin, HOMA-IR, TG, or total cholesterol and HDL-cholesterol levels. Moreover, the area of sdLDL was significantly higher in the *T/T *homozygotes (1.7 ± 3.4 vs. 0.8 ± 1.9%). There was no difference in the area of sdLDL between subjects with and without obesity (as BMI of more than 25 kg/m^2^).

**Table 2:** A stepwise multiple regression analysis for small dense low-density lipoprotein

Independent variables	Regression coefficient (95% CI *)		P value
Fasting plasma glucose (mg/dL)	0.013	(from [0.003] to [0.024])	0.015
Sex	0.774	(from [0.118] to [1.431])	0.021
*Clock 3111 T/C *SNP ^a^	-0.838	(from [-1.598] to [-0.077])	0.031

* CI indicates coefficient interval.

^a ^*Clock 3111 *SNP was scored as follows: *T/T *type = 0, *C/T *or *C/C *type = 1.

In the questionnaire on lifestyle, there was no significant difference in sleeping time between carriers and non-carriers of the *C *allele (Table 2). Moreover, a stepwise multiple regression analysis for the area of sdLDL revealed a significant, negative, and independent association of the *Clock 3111 T/C *SNP (β = -0.114, p = 0.031) (Table 3).

## Discussion

The present study is the first to demonstrate an association between the *Clock 3111 T/C *SNP and sdLDL in the circulation among community-dwelling people. The most important finding is that the *Clock 3111 T/T *homozygous form might increase the area of sdLDL, independently of potential confounding factors. The mechanism by which the clock molecule and the variation in its gene could be associated with sdLDL remain to be elucidated. The *Clock *mutant mouse has altered feeding patterns accompanied by the development of obesity and elevated TG, glucose and leptin levels [11], and mutations in *Clock *and *Bmal *influence the diurnal variation in TG and glucose concentrations [31]. The *Clock *mutant mice is also both hyperphageic and obese, and exhibits abnormalities in circulating levels of glucose, lipids, and hormones/adipokines, consistent with a cardiometabolic syndrome phenotype [32]. It was reported, however, that the *3111 T/C *SNP does not alter the amino acid sequence of the clock protein because *3111 T/C *SNP locates in the 3'-flanking region of the *Clock *gene. Therefore, we speculate that the *3111 T/C *SNP may affect *Clock *mRNA level, helping to alter clock protein levels and thereby disturbing other clock molecules' network and circadian rhythmicity. The *3111 T/C *SNP has been associated with sleep disruptuin in humans in many studies [13,16,33-38] but not all [39]. Further examination is needed to clarify the mechanism underlying the association between the *3111 T/C *SNP and sdLDL.

There are several limitations to our study. First, no assessment of sleep patterns (sleep onset time, wake time, and daytime sleepiness) was performed, although there was no significant difference in the prevalence of skipping breakfast and sleeping time. Second, only one SNP was examined. It has been reported that the haplotypes of rs1554483G and rs4864548A are associated with a 1.8-fold increase in the risk of overweightness or obesity [40]. So, we would need to consider more studies on the association between *3111 T/C *tag and other variants. Third, considering the small sample size in our study, we need to investigate the replication in independent cohorts to confirm our observed association. In the light of our findings, it will be important to further establish the effects of the *3111T/C *SNP on gene regulation.

## Conclusion

In conclusion, our findings indicate the *3111T/C *SNP of the *Clock *gene might modify the existence of sdLDL in the circulation. Understanding the mechanisms underpinning the relationship between the environment, our circadian rhythms, and dyslipidemia in the development of cardiovascular disease will provide important pathways for both prevention and management of these conditions.

## List of abbreviations

LDL: low-density lipoprotein; sdLDL: small dense LDL; TG: triglycerides; HDL: high-density lipoprotein; SNP: single nucleotide polymorphism; Clock: circadian locomotor output cycles protein kaput; BMAL1: brain and muscle Arnt-like protein 1; BMI: body mass index; CAD: coronary artery disease; HOMA-IR: homeostasis model assessment of insulin resistance.

## Competing interests

The authors declare that they have no competing interests.

## Authors' contributions

SF has been the project's reader; KT, KK, YS, SF, and NS have participated in critical revision of this manuscript; KT has performed in the statistical analysis; All authors have read and approved the final version of this manuscript.

## Pre-publication history

The pre-publication history for this paper can be accessed here:

http://www.biomedcentral.com/1471-2350/11/150/prepub
